# Vericiguat: The Fifth Pillar of Heart Failure

**DOI:** 10.31083/RCM50913

**Published:** 2026-06-16

**Authors:** Ram B Singh, Najah R Hadi, Abdulla Shehab, Galal Elkilany

**Affiliations:** ^1^Department of Medicine, Halbeg Hospital and Research Institute, 244001 Moradabad, India; ^2^Faculty of Medicine, University of Kufa, 54001 Najaf, Iraq; ^3^Department of Cardiovascular Medicine, UAE University, 19666 Al-Ain, UAE; ^4^Medical Departments, Sentara Northern Virginia Medical Center, Woodbridge, VA 22191, USA

## 1. Introduction

Despite increased availability of drugs for treatment of heart failure (HF), the Four Pillar Directed Therapy (FPDT) does not provide absolute protection in its treatment [1, 2, 3, 4, 5]. Interestingly, contemporary therapy of HF emphasizes early initiation and rapid up-titration of four classes of basic drugs, including angiotensin converting enzyme (ACE)/receptor blockers or angiotensin receptor-neprilysin inhibitors (ARNI), sodium-glucose cotransporter 2 (SGLT2) inhibitors, β blockers, mineralo-corticoid receptor antagonists (MRA), and with diuretics for the relief of symptoms due to volume overload [3, 4, 5]. However, it is possible that guideline directed medical therapy (GDMT) may not provide absolute protection in a subgroup of patients with heart failure, indicating the need for a fifth drug, vericiguat, which is a key oral soluble guanylate cyclase (sGC) stimulator [5, 6, 7]. Vericiguat may provide further benefits among high-risk patients with worsening HF with reduced ejection fraction (HFrEF) [6, 7]. Recent pooled analysis of the VICTORIA and VICTOR trials provides compelling evidence that vericiguat reduces mortality in patients with HFrEF, suggesting that, as a foundational fifth pillar of GDMT, it is distinct from the existing four [7]. This communication emphasizes that the Five Pillars of HF, including vericiguat as the fifth Pillar, may improve cardiovascular outcomes, including cardiovascular (CV) and all-cause mortality, as well as HF hospitalization.

In issue 4 of this journal in 2026, a review emphasizes that GDMT, along with vericiguat, may significantly improve survival and quality of life in patients with both reduced and preserved ejection fraction (HFpEF) [8]. Despite advances in the management of HF, it remains a leading cause of morbidity and mortality, partly due to the suboptimal implementation of GDMT [2, 3, 4, 5]. Poor adherence to pharmacological therapy may result from therapeutic inertia and concerns about hypotension during treatment [1, 2, 3]. In addition, it is also possible that other mechanisms of heart failure are not fully addressed by GDMT. Approximately half of all cases of HF are attributed to decreased left ventricular systolic function, known as HFrEF. However, mortality rates are similar between HFrEF and HFpEF.

## 2. Effects of Vericiguat in Heart Failure

Vericiguat, formerly indicated solely for patients with low ejection fraction, is now recommended for all patients with HF by most of the agencies, including the International College of Cardiology [3, 4, 5, 6, 7]. Efficacy in HFmrEF supports this medication’s promotion to class IIB recommended status for this population. These medications include ACE inhibitors, ARBs or ARNIs, beta-blockers, SGLT2 inhibitors, and mineralo-corticoid receptor antagonists (MRAs) [5]. In the recent VICTOR trial, performed at 482 centers across 36 countries, among patients (n = 6105, median age 68.0 years, 4665 (76.4%) were males), with HFrEF (≤40%), 3053 patients were randomly assigned: to vericiguat and 3052 to the placebo group, 1440 (23.6%) patients were females [6]. After a median follow-up of 18.5 months, primary outcome events were similar between the vericiguat group and the control group (549 [18.0%] vs 584 [19.1%]). Since the primary outcome was not statistically significant, as mentioned in the study design, all the findings of the secondary and exploratory outcomes are nominal. Interestingly, cardiovascular mortality did not differ significantly between the intervention and control groups (292 [9.6%] vs 346 [11.3%]; HR 0.83). Hospitalizations for HF were also similar between the two groups (348 [11.4%] vs 362 [11.9%]; HR 0.95).

No significant differences in all-cause mortality were observed between the intervention group and control groups (377 [12.3%] vs 440 [14.4%]; HR 0.84) [6]. Adverse effects were also similar between groups. Thus, the VICTOR trial’s primary outcome was neutral; it did not show a benefit for the composite endpoint of CV death or HF hospitalization.

### Vericiguat: The Fifth Pillar of Heart Failure

Recently, a pooled analysis was conducted of the VICTORIA and VICTOR trials to provide compelling data that vericiguat reduces mortality in HFrEF, indicating that it should be considered a foundational fifth pillar of GDMT. The VICTOR trial was neutral for the primary endpoint, but its nominally significant mortality benefit made it consistent with the signal observed in the pooled analysis [6]. The individual participant data analysis of the VICTORIA (n = 5050) and VICTOR (n = 6105) trials (total n = 11,155) showed that vericiguat consistently decreases the risk of cardiovascular death or hospitalization for HF in patients with HFrEF across a wide spectrum of risk factors [7]. The treatment benefit was consistent regardless of the risk profile, with notable efficacy seen in patients with NT-proBNP ≤6000 pg/mL. The primary endpoint shows that vericiguat significantly decreased the combined outcome of cardiovascular mortality or hospitalization due to heart failure. Benefits were also observed for each component: cardiovascular death and heart failure hospitalizations, as well as all-cause deaths. The drug was also effective across various risk profiles, providing a consistent treatment effect without significant inter-trial heterogeneity. Vericiguat was well-tolerated, with a 89.2% tolerance rate for the 10 mg dose after one year in the pooled analysis. The results indicate that vericiguat is a viable therapeutic option for decreasing CV events in both high-risk (post-hospitalization) and lower-risk, stable HFrEF patients [7]. The study concluded that the beneficial effects of vericiguat in HFrEF are consistent across a broader, less selected population of patients, having an independent mechanism of action, indicating that it could be considered as the Fifth Pillar of HF management.

Since diuretics are insufficient to control volume overload in many patients with HF, this emphasizes the need for developing novel medicinal approaches to treating HF, which may be the Fifth Pillar of HF therapy. Potential therapies for HFrEF have recently been examined, including sGC agonists and other members of a novel family of medicines that target various portions of the cyclic guanosine monophosphate (cGMP) pathway [9]. In a study with a mean follow-up of 10.8 months, vericiguat substantially decreased the risk of the main composite endpoint, which was composed of cardiovascular mortality and the first hospitalization for HF (35.5% vs 38.5%, *p* = 0.02) [10]. These findings revealed that the decline in HF hospitalizations was the main contributor to the difference, although deaths from cardiovascular causes were almost the same in both groups. It is of interest that although this agent did not reduce the composite endpoint in one trial [6], it is still a candidate for a pillar, possibly because it targets a different pathway and provides a mortality benefit with safety when the results of the trial are combined with another more recent trial [7].

**Fig. 1.**
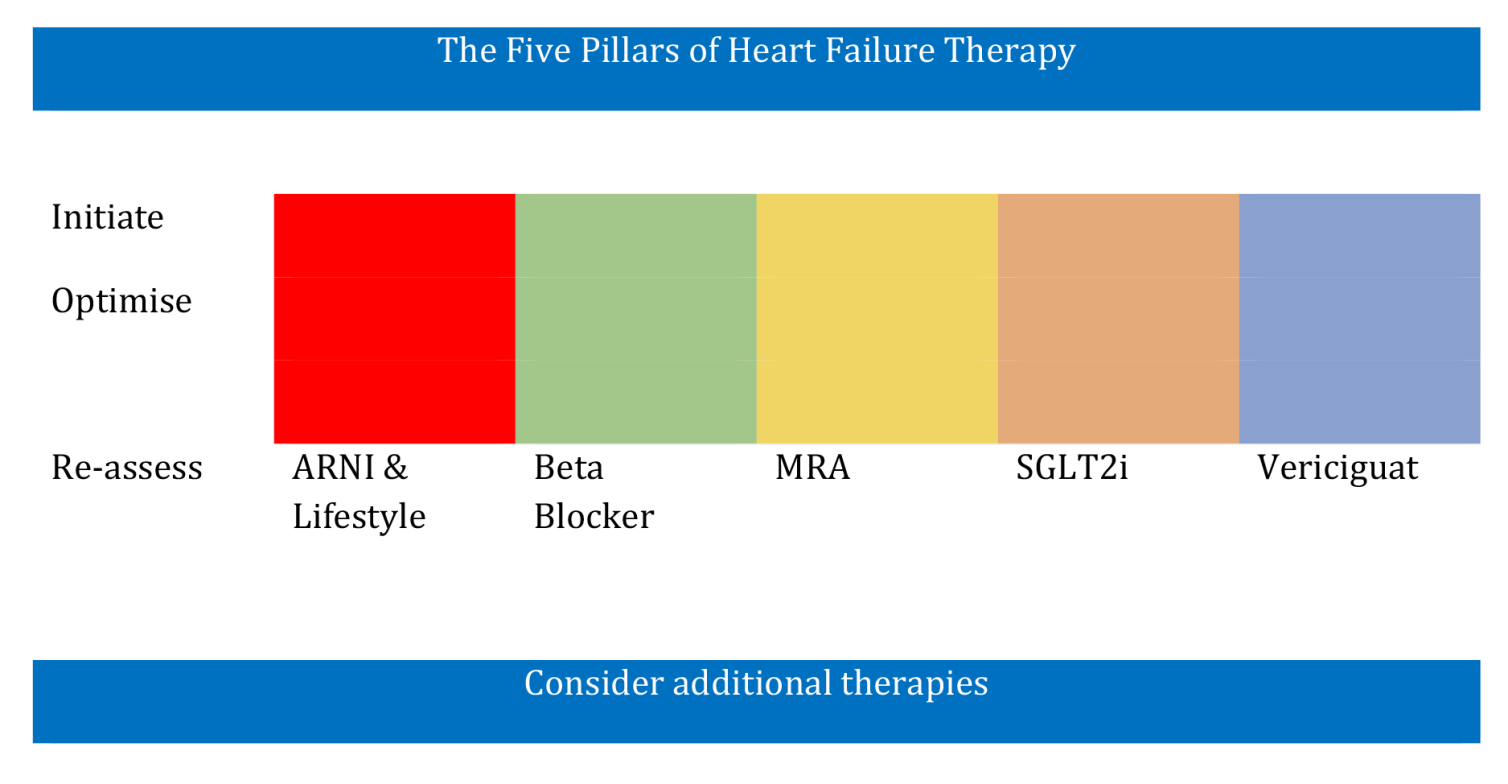
**Five pillars of heart failure management**. ARNI, angiotensin receptor-neprilysin inhibitor; MRA, mineralocorticoid receptor antagonist; SGLT2i, sodium-glucose co-transporter 2 inhibitors.

The role of the “four pillars” of HF therapy, including beta blockers, ARNI/angiotensin receptor blockers (ARBs), MRAs, and sodium-glucose co-transporter 2 Inhibitors (SGLT2i), is established [11], but some patients continue to die or remain uncontrolled during the management of HF [1, 2, 3]. Therefore, it may be proposed that vericiguat could be the Fifth Pillar of HF (Fig. 1, Ref. [1]).

It is possible that in the future, methods to improve outcomes may be the integration of multiomic profiling involving personalized medicine and innovative designs of clinical trials such as chronotherapy, to address residual risk for identification of new therapeutic targets.

## 3. Conclusions

The Four Pillars of heart failure include SGLT2i, which is also recommended for HFpEF. Other pillars of heart failure are ACE inhibitors and ARBs, or ARNIs, along with beta-blockers and MRAs. ARNIs are preferred because of their independent mechanism of selective action in heart failure. Vericiguat may be the Fifth Pillar of heart failure because it has a unique mortality benefit, and it also has an independent mechanism of action. Therefore, its potential beneficial role calls for an update to the current GDMT.
